# Evaluating the Performance of State-of-the-Art Artificial Intelligence Chatbots Based on the WHO Global Guidelines for the Prevention of Surgical Site Infection: Cross-Sectional Study

**DOI:** 10.2196/75567

**Published:** 2025-07-31

**Authors:** Tianyi Wang, Ruiyuan Chen, Baodong Wang, Congying Zou, Ning Fan, Shuo Yuan, Aobo Wang, Yu Xi, Lei Zang

**Affiliations:** 1 Beijing Chao-Yang Hospital, 5 JingYuan Road, Shijingshan District, Beijing, 100043, China, 86 51718268

**Keywords:** artificial intelligence, large language model, natural language processing, global guideline, health communication, surgical site infection

## Abstract

**Background:**

Surgical site infection (SSI) is the most prevalent type of health care–associated infection that leads to increased morbidity and mortality and a significant economic burden. Effective prevention of SSI relies on surgeons strictly following the latest clinical guidelines and implementing standardized and multilevel intervention strategies. However, the frequent updates to clinical guidelines render the processes of acquisition and interpretation quite time-consuming and intricate. The emergence of artificial intelligence (AI) chatbots offers both possibilities and challenges to address these issues in the surgical field.

**Objective:**

This study aimed to test the multidimensional capability of state-of-the-art AI chatbots for generating proper recommendations and corresponding rationales concordant with the global guideline for the prevention of SSI.

**Methods:**

Referred by other authoritative guidelines, recommendations and corresponding rationales from the 2018 World Health Organization global guidelines were refined and selected as benchmarks. Then, they were rephrased into a combined format of closed-ended queries for recommendations and open-ended queries for corresponding rationales, whereafter input into ChatGPT-4o (OpenAI), OpenAI-o1 (OpenAI), Claude 3.5 Sonnet (Anthropic), and Gemini 1.5 Pro (Google) 3 times. All responses were individually evaluated in 10 evaluation metrics based on the QUEST dimensions by 4 multidisciplinary senior surgeons using a 5-point Likert scale. The multidimensional performances among chatbots were compared, and the interrater agreements were calculated.

**Results:**

A total of 300 responses to 25 queries were generated by the 4 chatbots. The interrater agreements of the evaluators ranged from moderate to good (0.54‐0.87). In response to recommendations, the average accuracy, consistency, and harm scores for all chatbots were 4.03 (SD 1.09), 4.07 (SD 0.88), and 4.29 (SD 1.01), respectively. In responses for rationales, 4 subdimensions, including harm (mean 4.22, SD 0.97), relevance (mean 4.15, SD 0.83), fabrication and falsification (mean 4.12, SD 1.02), and understanding and reasoning (mean 4.04, SD 0.92) averagely scored ≥4. In contrast, consistency (mean 3.94, SD 0.72), clarity (mean 3.94, SD 0.89), comprehensiveness (mean 3.85, SD 0.83), and accuracy (mean 3.74, SD 0.91) performed at a moderate level. For the whole responses, the average self-awareness and trust and confidence scores for all chatbots were 3.84 (SD 0.89) and 3.88 (SD 0.91), respectively. Based on the average scores of the subdimensions, Claude 3.5 Sonnet and ChatGPT-4o were the top 2 outperformed models.

**Conclusions:**

The performance of AI chatbots in providing responses regarding well-established global guidelines in the prevention of SSI was acceptable, demonstrating immense potential in clinical applications. Nonetheless, a critical issue is the necessity of enhancing the stability of chatbots, as inaccurate responses can lead to severe consequences for SSI. Despite its limitations, it is anticipated that AI will trigger far-reaching changes in how clinicians access and use medical information.

## Introduction

Surgical site infection (SSI) is defined as the postoperative infection of the incision, organ, or space, which is the most prevalent type of health care–associated infection [1,2]. The pooled incidence of SSI among patients achieves 2.5% globally, leading to increased morbidity and mortality and a significant economic burden [3]. Therefore, the prevention of SSI has consistently been a focal and challenging task in the field of surgery, which requires surgeons to take standard and multidimensional approaches throughout the entire perioperative period following specific clinical guidelines. Several agencies and professional organizations have developed guidelines for the prevention of SSI [1,2,4-7]. However, certain problems still exist. For example, there are discrepancies in guidelines formulated by various institutions, and the guidelines are continually updated. Thus, it is challenging for surgeons, especially junior surgeons, to find a concise and reliable source to catch up with the integrated and up-to-date standards for the prevention of SSI in routine clinical practice. Besides, there is a potential demand for patients undergoing surgeries to conveniently seek additional credible information about SSI other than from their physicians.

The emergence of artificial intelligence (AI) chatbots based on large language models (LLMs) offers both possibilities and challenges to address these issues [8,9]. The LLMs are generally trained by a massive open-source corpus from various domains online to flexibly generate human-like responses to prompts on command. The versatility of chatbots rapidly captured unprecedented attention in the medical field because of their potential to lead a revolution of medical information acquisition mode from traditional static searching to dynamic AI-backed knowledge gathering [10]. However, this emerging technique also comes with some defects, such as misunderstanding of prompt, lack of self-awareness, fabrication, falsification, or plagiarism [11-13], which are always magnified in health care practice as it may considerably aggravate health and economic burden. Therefore, the health care uses of chatbots have been widely assessed in several disciplines of medicine and have gained promising results [9,11]. However, previous studies failed to follow a well-developed principle to evaluate the capability of LLMs comprehensively. Besides, to the best of our knowledge, there is no study investigating the performance of current chatbots in providing specialized medical guidance for the prevention of SSI, which hinders our understanding of how it could potentially alleviate surgeons’ information retrieval and decision-making burdens in this field.

QUEST, the first comprehensive and practical framework for the human evaluation of LLMs in health care, was developed in September 2024 [11]. In total, 5 evaluation principles were covered in QUEST, including quality of information, understanding and reasoning, expression style and persona, safety and harm, and trust and confidence.

In accordance, this study aimed to test the multidimensional capability of 4 state-of-the-art AI chatbots for generating proper recommendations and corresponding rationales concordant with the World Health Organization (WHO) global guideline for the prevention of SSI.

## Methods

### Overview

**Figure 1.**
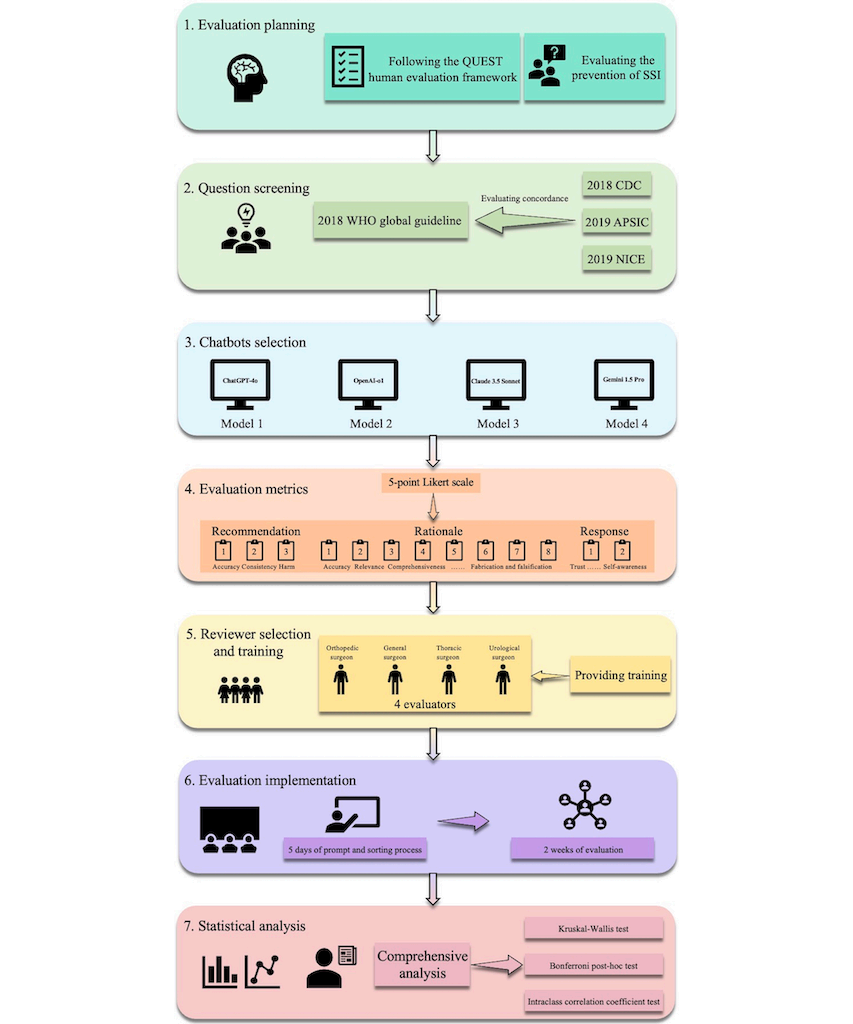
Flowchart of overall study design. APSIC: Asia Pacific Society of Infection Control; CDC: Centers for Disease Control and Prevention; NICE: National Institute for Health and Care Excellence; SSI: surgical site infection; WHO: World Health Organization.

This cross-sectional study was performed following the QUEST human evaluation framework [11] (Figure 1) and the Strengthening the Reporting of Observational Studies in Epidemiology (STROBE) reporting guideline (Checklist 1) [14].

### Guidelines and Recommendations Selection

Various guidelines have been developed to provide recommendations for the prevention of SSI. In consideration of both authority and currency, this study enrolled 4 guidelines for initial benchmarks, including 2018 WHO global guideline [1], 2017 Centers for Disease Control and Prevention (CDC) guideline [2], 2019 Asia Pacific Society of Infection Control guideline [4], and 2019 National Institute for Health and Care Excellence guideline (updated in August 2020) [5]. The WHO global guideline for the prevention of SSI was further chosen as the primary criterion for 2 main reasons. First, it is the only generalized guideline focusing on the global population, and the recommendations were developed by referring previous guidelines [15]. Besides, a specific rationale is attached to each recommendation.

All 34 recommendations of 26 sections in the WHO global guideline were extracted as benchmark candidates and compared with the corresponding recommendations in other guidelines. The concordance was evaluated by a grading scale (0‐3): 1=perfectly coincident, 2=reconcilable, 3=contradictory, and 0=not mentioned. The assessment was conducted by 2 surgeons independently (TW, with 5 y of experience and NF, with 11 y of experience), and discordant results were resolved through discussion to reach a consensus. The exclusion criteria were (1) recommendations mentioned in none of the other guidelines, and (2) recommendations conflicting with another guideline. Finally, 25 recommendations in 20 sections were determined as the standardized benchmarks. The details of the recommendation selection are illustrated in Multimedia Appendix 1, and the flowchart of the selection procedure is illustrated in Multimedia Appendix 2.

### Chatbots Selection and Prompt Strategy

Forrester Research, a leading market research and consulting company, recently issued the Forrester Wave report for artificial intelligence foundation models for language (AI-FMLs) in the second quarter of 2024 and determined the top-10 AI-FMLs in view of current offering, strategy, and market presence [16]. In this study, 4 state-of-the-art LLMs were further selected and investigated: ChatGPT-4o (OpenAI), OpenAI-o1 (OpenAI), Claude 3.5 Sonnet (Anthropic), and Gemini 1.5 Pro (Google). The main features of these LLMs are detailed in Multimedia Appendix 3.

Each recommendation was rephrased as a closed-ended question. Meanwhile, an open-ended question for the rationale of the recommendation was also proposed to test the models’ reliability and logical reasoning. The 2 questions were synthesized into a single sentence using the structured prompt as follows: “For the prevention of surgical site infection, [Query]? Provide the recommendation and the rationale for the recommendations.” Each prompt was input into a new window in each chatbot model 3 times, and the answers were recorded verbatim. Since current chatbots may tend to provide long-winded and confounding responses, all answers were manually divided into recommendations and the corresponding rationales for further separate analysis. The prompt and sorting process was performed by 1 surgeon (RC, with 3 y of experience) with a 5-day course in November 2024. All prompts based on the same recommendation were repeated on the same day, thereby minimizing the impact of temporal intervals.

### Performance Evaluation

The answers to the recommendations and rationales from chatbots were compared with the standardized benchmarks, respectively. The evaluation metrics were determined based on the QUEST dimensions, which are designed for human evaluations accommodating the diverse subdomains in health care comprehensively [11]. For recommendations prompted by closed-ended questions, 3 dimensions, including accuracy, consistency, and harm, were assessed. For rationales prompted by open-ended questions, eight dimensions, including (1) accuracy, (2) relevance, (3) comprehensiveness, (4) consistency, (5) understanding and reasoning, (6) clarity, (7) harm, and (8) fabrication and falsification, were assessed. With regard to the whole answer containing both recommendations and rationales, we further evaluated the (1) self-awareness and (2) trust and confidence of the LLMs. All dimensions were evaluated using the 5-point Likert scale, and based on the response of the first prompt for each LLM (except consistency). Consistency was assessed by comparing the answers of 3 repeated prompts. The elaboration of the 5-point Likert scale was presented in Multimedia Appendix 4.

The responses were evaluated and scored by 4 senior surgeons in different subspecialties with a 2-week course (evaluator 1, an orthopedic surgeon with 31 y of experience; evaluator 2, a general surgeon with 25 y of experience; evaluator 3, a thoracic surgeon with 22 y of experience; and evaluator 4, a urological surgeon with 26 y of experience) independently. Before the evaluation implementation, all surgeons were asked to thoroughly familiarize themselves with the evaluation checklist and standard recommendations and rationales. During the review procedure, the surgeons were blinded to the answer source, and the average of the 4 surgeons was calculated as the final score for each item. The reliability among surgeon evaluations was determined by calculating interrater agreement between the raters.

### Statistical Analysis

Statistical analysis was performed using SPSS 24.0 (IBM). Due to the equidistance of the 5-point Likert scale, all data were presented as mean (SD). Comparison for grading among LLMs was performed using the Kruskal-Wallis test, followed by the Bonferroni post hoc test. The Chi-square tests or Fisher exact tests, followed by the Bonferroni post hoc test, were used to compare categorical variables. Interrater agreement was assessed by the intraclass correlation coefficient (ICC) test for absolute agreement. The ICCs were determined as excellent (ICC≥0.90), good (0.75≤ICC<0.90), moderate (0.50≤ICC<0.75), and poor (ICC<0.50). Statistical difference was set at *P*<.05, whereas significant statistical difference was set at *P*<.01.

### Ethical Considerations

Ethical approval for this study was not required as all chatbots used were public resources, and no human or animal data were involved.

## Results

A total of 300 responses, containing 100 first-time responses, to 25 queries were generated by the 4 chatbots and evaluated by 4 senior surgeons. All responses are listed in Multimedia Appendix 5. The overall interrater agreements of the evaluators ranged from moderate to good. The ICCs were 0.87 (0.78‐0.93) for recommendation-related evaluations, 0.79 (0.48‐0.89) for rationale-related evaluations, 0.54 (0.40‐0.66) for self-awareness, and 0.62 (0.47‐0.74) for trust and confidence.

**Figure 2.**
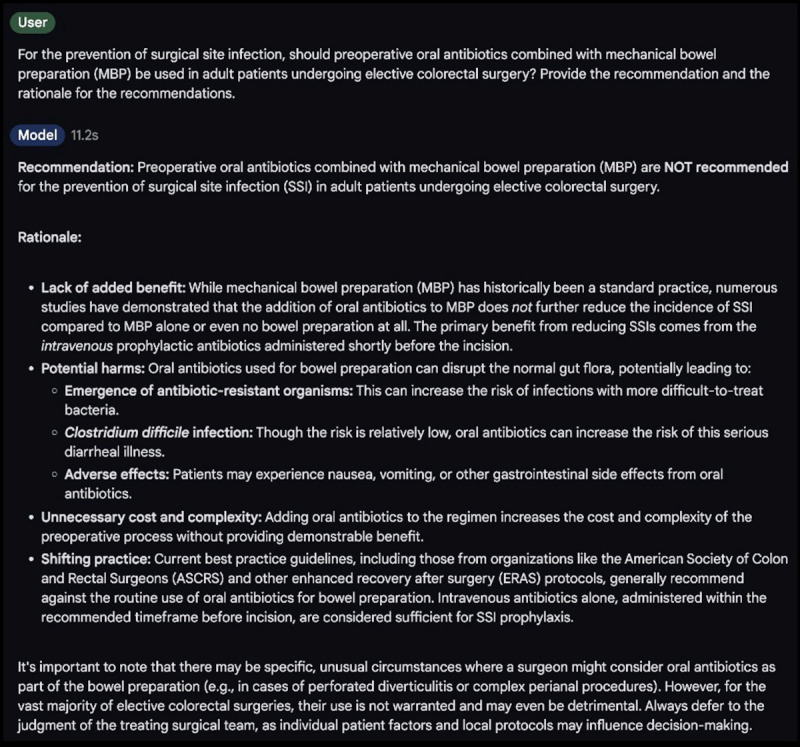
Example of a completely inaccurate response from Gemini 1.5 Pro.

**Figure 3.**
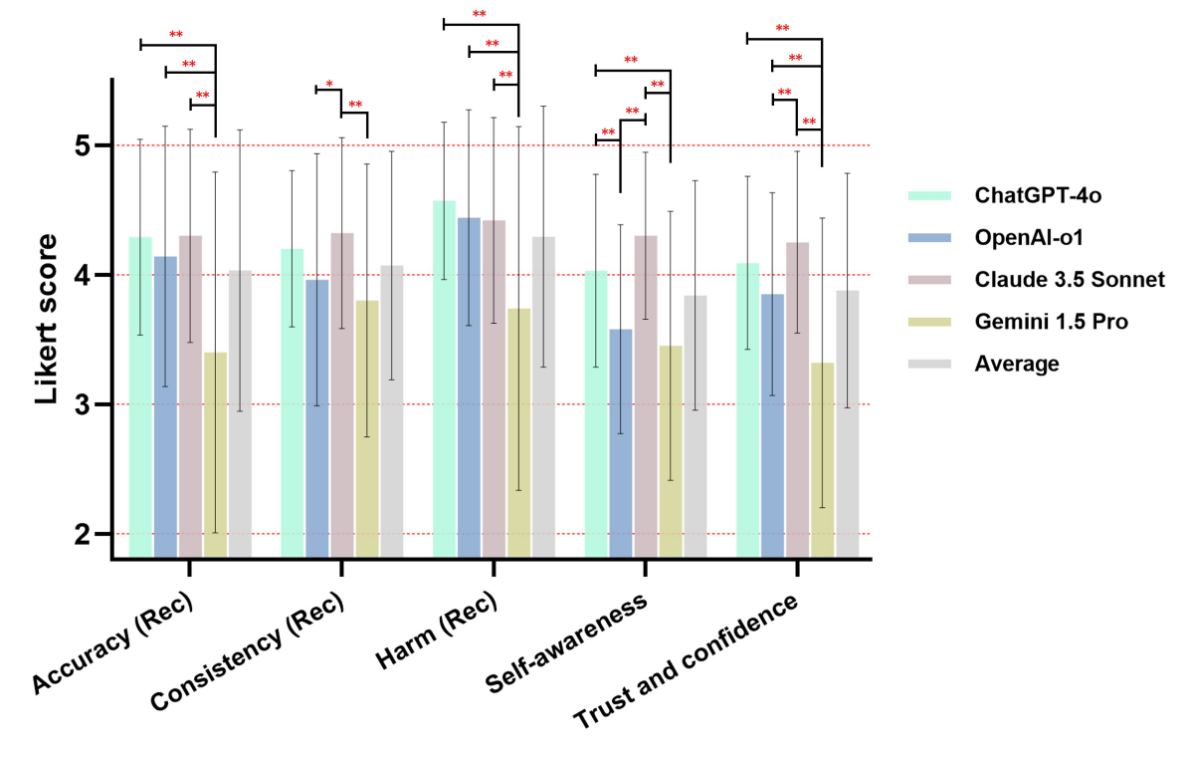
Performance results of the chatbots in providing recommendations and whole responses. Rec: recommendation. * indicates statistical difference, and ** indicates significant statistical difference.

The score for each item (response to query 1‐25) was determined by averaging scores from 4 evaluators (integers ranging from 1 to 5), and the final score for each evaluation dimension was then calculated by the average of all items. In responses for recommendations, the average accuracy score achieved 4.03 (SD 1.09) among models. ChatGPT-4o, OpenAI-o1, and Claude 3.5 Sonnet were scored at high levels of accuracy (4.14‐4.30). The accuracy score of Gemini 1.5 Pro was 3.40 (SD 1.39), significantly lower than the others (adjusted *P*<.01). An example of a completely inaccurate response from Gemini 1.5 Pro is provided in Figure 2. The average consistency across 3-time responses for recommendation was 4.07 (SD 0.88). Claude 3.5 Sonnet achieved the highest score of 4.32, followed by ChatGPT-4o (4.20). Claude 3.5 Sonnet outperformed OpenAI-o1 (vs 3.96, adjusted *P*=.04) and Gemini 1.5 Pro (vs 3.80, adjusted *P*<.01). The harmfulness of the LLMs responses for recommendations was the best-performed dimension, with an average score of 4.29 (SD 1.01), whereas Gemini 1.5 Pro was more likely to generate harmful recommendations, with a score of 3.74 (SD 1.40) than the others (adjusted *P*<.01; Figure 3, Multimedia Appendix 6).

The self-awareness of the whole responses was scored at a moderate level (mean 3.84, SD 0.89). Claude 3.5 Sonnet (4.30) and ChatGPT-4o (4.03) performed well in recognition of limits, better than OpenAI-o1 (vs 3.58, both adjusted *P*<.01) and Gemini 1.5 Pro (vs 3.45, both adjusted *P*<.01). The overall evaluator’s subjective trust and confidence in LLMs was also moderate, scoring at 3.88 (SD 0.91). In subgroup comparison, Claude 3.5 Sonnet was the most trusted LLM (4.25), followed by ChatGPT-4o (4.09) and OpenAI-o1 (3.85). Experts showed significantly less confidence with Gemini 1.5 Pro (3.32) than other models (adjusted *P*<.01; Figure 3, Multimedia Appendix 6).

**Figure 4.**
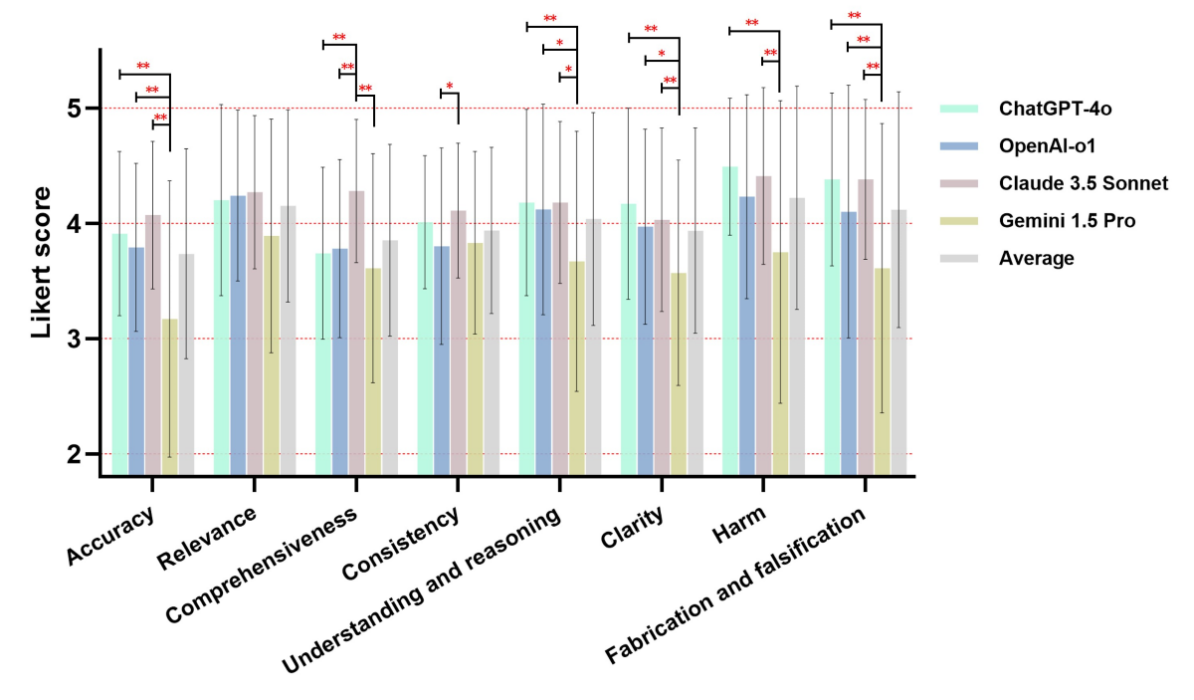
Performance results of the chatbots in providing rationales. * indicates statistical difference, and ** indicates significant statistical difference.

Regarding the responses for rationales, 8 dimensions were further assessed. Furthermore, 4 subdimensions, including harm (mean 4.22, SD 0.97), relevance (mean 4.15, SD 0.83), fabrication and falsification (mean 4.12, SD 1.02), and understanding and reasoning (mean 4.04, SD 0.92), were averagely scored≥4. Whereas, consistency (mean 3.94, SD 0.72), clarity (mean 3.94, SD 0.89), comprehensiveness (mean 3.85, SD 0.83), and accuracy (mean 3.74, SD 0.91) were performed at a moderate level. Similar to the recommendation, Claude 3.5 Sonnet and ChatGPT-4o were the top 2 outperformed models. The details of the performance among LLMs are illustrated in Figure 4 and Multimedia Appendix 6. Furthermore, 55 (55%) rationales provided specific authoritative guidelines or citations, with 23 (42%) originating from Claude 3.5 Sonnet, 18 (33%) from OpenAI-o1, 8 (15%) from ChatGPT-4o, and 6 (11%) from Gemini 1.5 Pro.

Within 25 trios of answers generated by each model, the unsatisfactory performance (Likert score <3) accounting for subdimensions in the whole response and recommendation was further identified. There were 12 responses that performed poorly in self-awareness, with 8 (67%) by Gemini 1.5 Pro, 3 (25%) by OpenAI-o1, and 1 (8%) in ChatGPT-4o. Gemini 1.5 Pro generated 8 (73%) responses with poor evaluators’ trust and confidence, followed by 2 (18%) in OpenAI-o1 and 1 (9%) in Claude 3.5 Sonnet. A total of 14 recommendation-related inaccurate responses were recognized, with the highest portion generated by Gemini 1.5 Pro (8/14, 57%), followed by OpenAI-o1 (4/14, 29%), and ChatGPT-4o and Claude 3.5 Sonnet (both 1/14, 7%). Of the total, 8 responses posed potential harmful recommendations, including 6 (75%) by Gemini 1.5 Pro and 1 (13%) by OpenAI-o1 and Claude 3.5 Sonnet, respectively. Only OpenAI-o1 (4/7, 57%) and Gemini 1.5 Pro (3/7, 43%) were attributed to 7 inconsistent trios of recommendations.

**Figure 5.**
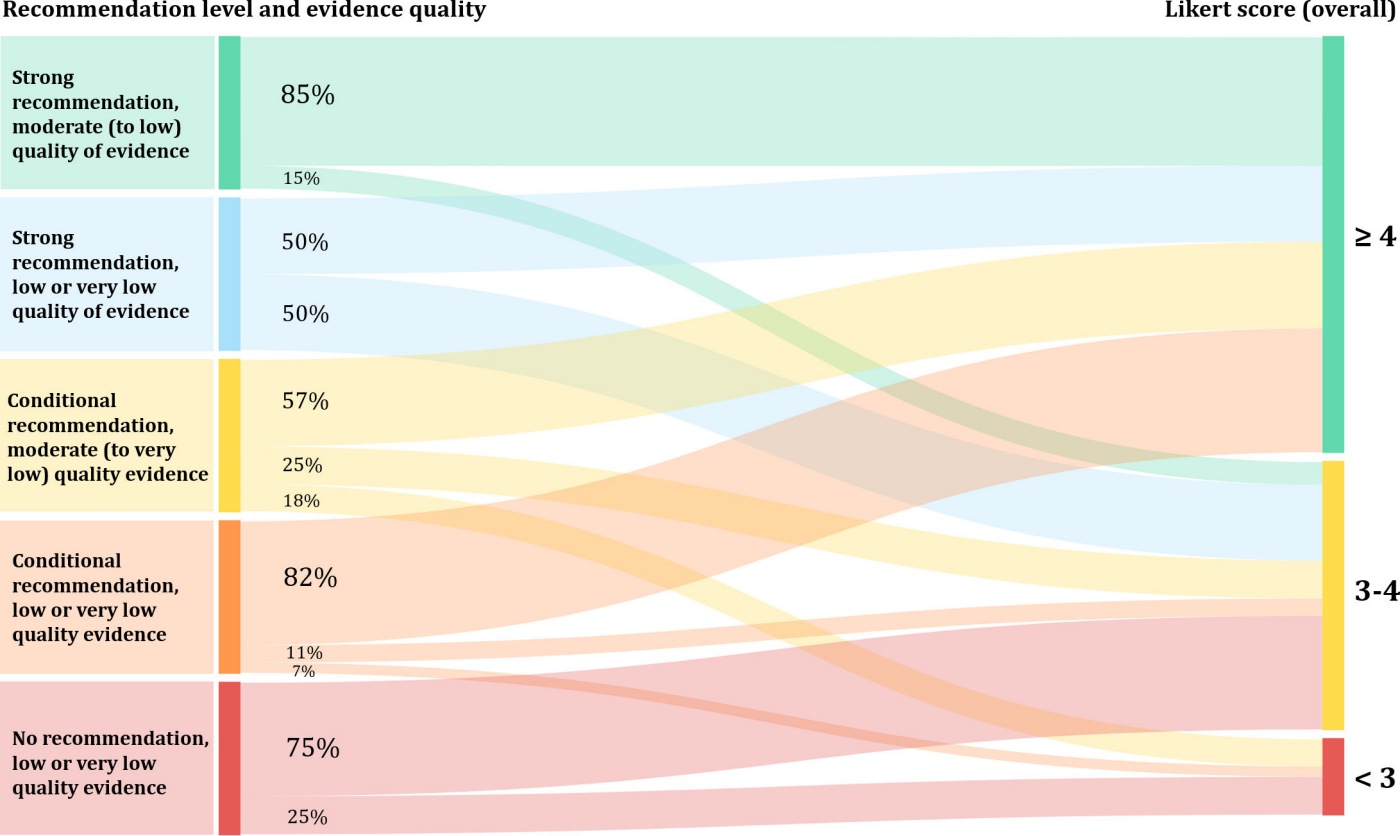
Sankey diagram showing the relationship between the recommendation level, evidence quality, and the performance of the model’s response.

The relationship between the recommendation level, evidence quality, and the performance of the model’s response is illustrated in Figure 5. With regard to strong recommendations in the guideline, 50%‐85% responses generated by LLMs were at high levels of performance (overall Likert score >4), and no response was scored <3. While 57% (16/28)‐82% (36/44) responses of LLMs were also scored >4 for conditional recommendations, 7% (3/44)‐18% (5/28) poor responses (scored <3) were observed. For the query with no recommendation in the guideline, LLMs were likely to generate more responses scored <3 (1/4, 25%) and fewer responses scored >4 (0/4,0), compared with strong or conditional recommendations (both adjusted *P*<.05). However, no relationship was identified between evidence quality and model’s response quality.

## Discussion

### Principal Findings

Effective prevention of SSI, a significant challenge in global public health, relies on surgeons strictly following the latest clinical guidelines and implementing standardized and multilevel intervention strategies. However, the frequent updates to clinical guidelines render the processes of acquisition and interpretation quite time-consuming and intricate. AI chatbots offer great potential for bridging that gap, and their applications in health care have shown promising results. However, their potential roles in the prevention of SSI remain inadequately explored. Therefore, this study conducted a cross-sectional analysis to systematically evaluate 4 state-of-the-art chatbots, including ChatGPT-4o, OpenAI-o1, Claude 3.5 Sonnet, and Gemini 1.5 Pro, and found their overall acceptable performances in the context of SSI prevention. The results emphasized the possibilities and limitations of the current AI chatbots in further triggering far-reaching changes in how clinicians access and use medical information.

The results indicated that the models performed best in terms of harm, relevance, fabrication, and falsification, while demonstrating relatively poorer performance in accuracy (regarding rationales), comprehensiveness, and self-awareness. The accuracy of the model is particularly critical in the medical domain. This study revealed that the average accuracy for recommendations and rationales was 4.03 (SD 1.09) and 3.74 (SD 0.91), respectively. Sciberras et al [17] also demonstrated the capability of an AI-based system to provide accurate answers (average score 3.87, SD 0.6) to real-world patient queries in inflammatory bowel disease. Similarly, Duey et al [18] found that ChatGPT could answer common questions about thromboembolic prophylaxis with reasonable accuracy compared with the North American Spine Society clinical guidelines.

Furthermore, other researchers have noted that another challenge faced by AI chatbots is inconsistency. Howard et al [19] reported that the clinical recommendations provided by ChatGPT often changed after repeated inquiries. Notably, the consistency of the recommendations (mean 4.07, SD 0.88) and the rationales (mean 3.94, SD 0.72) in this study was rated at a moderate to good level. Claude 3.5 Sonnet and ChatGPT-4.0 continued to be the top-performing models. Similarly, Samaan et al [20] and Yeo et al [21] found that consistency was high, with approximately 90% (137/151; 148/164) of all questions yielding 2 similar responses with comparable grading. Overall, our findings are consistent with previous research, indicating that the quality of AI chatbots in generating guideline-related responses is generally acceptable and holds promise for alleviating surgeons’ information retrieval and decision-making burdens for the prevention of SSI.

Another crucial issue is to clarify how different recommendation levels and evidence quality tiers in guidelines influence chatbot responses, which may provide additional insights into their limitation and generalizability in scenario-specific applications. Furthermore, it can guide the users regarding whether they should adjust inquiry strategies or degrees of confidence based on different recommendation levels and evidence quality when consulting chatbots. This study found that for questions with strong recommendations in the guideline, the responses generated by the LLMs exhibited high performance. Conversely, for questions that lacked recommendations in the guideline, the LLMs tended to produce a greater number of responses with scores <3 and fewer responses with scores >4. Furthermore, research by Shrestha et al [22] demonstrated that ChatGPT generally achieves high accuracy when addressing questions related to high-level recommendations in guidelines for low back pain. These findings may be attributed to the worrisome phenomenon known as “artificial hallucination,” wherein LLMs generate confident statements without support from training data [23]. OpenAI has explicitly acknowledged this issue, stating that “ChatGPT sometimes writes plausible-sounding but incorrect or nonsensical answers” [24]. However, for questions with conditional recommendations in the guideline, LLMs, particularly Gemini 1.5 Pro, are frequently prone to generating low-quality responses. These questions involve complex practical clinical considerations, presenting a significant challenge for LLMs, as the generated responses may exhibit uncertainty. Therefore, clinicians should comprehensively consider the complexity of the clinical problem, the clarity of the guidelines, and the variation in model performance when using LLMs to support decision-making.

Previous studies had focused on closed-ended questions and multiple-choice formats, which fail to adequately capture the complexity and nuances in medical decision-making [25]. Rationale is the inherently open-ended question that presents greater challenges because it reflects the logicality and complexity of both physicians and patients in clinical decision-making. In this study, we thereby designed a combination of closed- and open-ended questions and required the model to provide specific rationales to comprehensively assess its performance. Notably, we evaluated rationales based on the performance of recommendations and found that inaccurate recommendations adversely affected the accuracy, understanding and reasoning, harm, and fabrication and falsification of the rationales. Overall, the quality of the recommendations and the rationales in our study was rated at a moderate to good level, indicating that the models achieved a relatively satisfactory level of logicality. This suggests that the chatbots may be equipped to address various types of questions, thereby demonstrating broader applicability.

In contrast to other fields, medical practice cannot afford to rely on tools that occasionally provide erroneous answers, even if such instances are infrequent, as such inaccuracies can lead to catastrophic consequences [19,26,27]. It is thus crucial to straightforwardly identify the specific strengths and weaknesses in handling SSI-related queries among different models. This study showed that Gemini 1.5 Pro tends to produce more inaccurate and harmful responses than other models. The remaining 3 models generated only 2 unsatisfactory responses regarding harm and 6 concerning accuracy, which is both surprising and commendable. Figure 2 illustrates a typical erroneous case from Gemini 1.5 Pro. The panel suggests that preoperative oral antibiotics combined with mechanical bowel preparation should be used to reduce the risk of SSI in adult patients undergoing elective colorectal surgery, but Gemini 1.5 Pro fails to recommend this measure. This issue may stem from various factors, including insufficient or biased training data, misinterpretation of information, technical limitations, reliance on unreliable or outdated information sources, a lack of integration of medical expertise, and errors arising during programming or maintenance [28-31]. Consequently, surgeons and patients must exercise extreme caution when using chatbots to avoid erroneous medical decisions, as current chatbots do not always provide reliable information.

Furthermore, we found that a positive aspect of chatbots is their ability to recognize their processing patterns and the limitations and scope of their knowledge, including the need to reference the latest guidelines, provide disclaimers, and integrate clinical realities [11]. This study showed that 61% (61/100) of all responses exhibited a degree of self-awareness, with Claude 3.5 Sonnet (19/25, 76%) and ChatGPT-4.0 (18/25, 72%) outperforming Gemini 1.5 Pro (15/25, 60%) and OpenAI-01 (9/25, 36%) on this metric. Scaff et al [28] reported that AI chatbots, including ChatGPT 3.5, Microsoft Bing, Bard (Gemini; Google AI), and ChatGPT 4.0, provided disclaimers in 70% (21/30)‐100% (30/30) of responses. While this ensures optimal health care, it raises an important question: if the answers are neither sufficiently assertive nor clear, why do individuals continue to consult AI chatbots? Users expect accurate and specific answers supported by evidence [17], and future iterations of models should be designed to meet this expectation.

A significant challenge in assessing the accuracy of chatbot responses is their opaque decision-making processes, often described as the “black box” problem [28]. Although these models have made advances in improving the transparency of information retrieval, the disclosure of information sources remains limited, making it difficult for users to understand the basis of their responses. In this study, only 47% (47/100) of first responses provided specific reference guidelines and citations. Notably, Claude 3.5 Sonnet cited the WHO, CDC, and other authoritative guidelines as direct evidence in 23 of the 25 questions it answered, demonstrating its exceptional capability in extracting research related to the prevention of SSI. In contrast, Nwachukwu et al [32] found that only 10% (20/192) of the responses from ChatGPT-4, Gemini, Mistral-7B, and Claude-3 included specific references or provided links to accessible peer-reviewed materials to support their recommendations. This lack of information disclosure, especially the failure to provide specific sources when generating responses on particular topics, may impede users from effectively using AI chatbots for specific tasks that require evidence-based support. Another challenge to inquiring chatbots about medical problems is facing numerous ethical issues [33]. First, the training data for these models are mainly from Western medical literature and specific populations, leading to potentially biased output results [34]. Second, the attribution of responsibility when models generate erroneous medical advice remains ambiguous. Furthermore, privacy protection of sensitive information and data security issues are of great concern [34,35]. Addressing these issues necessitates collaborative efforts across various sectors, leveraging the synergistic interplay of technological, ethical, and legal frameworks to ensure that chatbots can serve all users in a fair, safe, transparent, and efficient manner.

### Limitations

There are some limitations in our study. First, considering the rapid advancement of AI technology, the results of this cross-sectional study may only reflect the model’s performance at specific time points. Therefore, caution must be exercised in interpreting these findings, acknowledging that the current capabilities of chatbots may differ from our results. Nevertheless, the insights gained from this study hold significant value and establish a foundation for future studies using more advanced models. Second, we did not investigate the impact of prompt engineering on responses. Prompts based on the same framework may yield different answers due to subtle variations in wording [36]. A prompt that proves effective for one model may not be suitable for another. Although a few recent studies and position papers have proposed strategies for formulating prompts [37-39], these have not yet been widely validated through extensive fact-checking. Thus, related studies should examine the applicability and robustness of prompts and continuously develop and update relevant guidelines. Third, the models evaluated were all closed-source commercially available models, which limits us to explain performance differences in detail from the models’ response generation techniques, training data, or model design. Furthermore, the methodology of the assessment for chatbot performance among the current studies was highly varied because there is no validated and widely recognized evaluation tool. As a result, it is hard to directly compare the quantitative results in this study with others. Besides, the evaluations (except for consistency) were totally based on the first response, which may not represent the LLM’s central tendency or range of performance, especially when the performance is inherently stochastic. Fourth, since there was only 1 surgeon from each subspecialty, we were unable to explore the potential impact of evaluators’ subspecialty knowledge on the scoring outcomes. Furthermore, the WHO global guidelines have not been updated since 2018 and may now be outdated, but they still represent the latest universally accepted organizational guidelines for SSI prevention. Since each LLM has a training data cutoff date and not all the latest research and clinical guidelines are publicly available and incorporated into the training (depending on the relationship between their publication time and the LLM’s training data cutoff), LLMs are not always up-to-date with the latest medical literature or clinical guidelines. Finally, while the number of questions included in this study is relatively small, they were selected based on the WHO global guidelines and refined by other authoritative guidelines strictly, ensuring that the chosen questions have the highest credibility. However, it may introduce potential selection bias that eliminates unique but valuable WHO recommendations from the evaluation. Future considerations should include updating guidelines and aligning clinical practices in specific regions.

### Conclusion

The performance of AI chatbots in providing responses regarding well-established global guidelines in the prevention of SSI was acceptable, demonstrating immense potential in clinical applications. In general, Claude 3.5 Sonnet and ChatGPT-4o were the top 2 outperformed models based on the average scores of the subdimensions. However, a crucial issue is the necessity for improving the stability of chatbots since inaccurate responses can lead to severe consequences for SSI. Despite its limitations, it is foreseeable that AI will trigger far-reaching changes in how clinicians access and use medical information. In the future, iterations of chatbots are expected to accelerate the dissemination and application of the latest medical knowledge, ensure the timeliness and scientificity of clinical decision-making, and propel health care services into a new era characterized by greater efficiency and precision.

## Supplementary material

10.2196/75567 Multimedia Appendix 1 The selection and refinement of the standardized benchmarks form WHO global guideline recommendations.

10.2196/75567 Multimedia Appendix 2 The flowchart of the recommendation selection procedure.

10.2196/75567 Multimedia Appendix 3 The main features of the four state-of-the-art LLMs used in this study.

10.2196/75567 Multimedia Appendix 4 Interpretation of the 5-point Likert scale system for evaluating response of LLMs.

10.2196/75567 Multimedia Appendix 5 All responses were generated by the four chatbots.

10.2196/75567 Multimedia Appendix 6 Comparison of the performances of chatbots’ responses for surgical site infection.

10.2196/75567 Checklist 1 STROBE (Strengthening the Reporting of Observational studies in Epidemiology) statement—checklist of items that should be included in reports of cross-sectional studies.

## Abbreviations

AI: artificial intelligence
AI-FMLs: artificial intelligence foundation models for language
CDC: Centers for Disease Control and Prevention
ICC: intraclass correlation coefficient
LLM: large language model
SSI: surgical site infection
STROBE: Strengthening the Reporting of Observational Studies in Epidemiology
WHO: World Health Organization
