# Supplementary figures and images for: Evaluating the Performance of State-of-the-Art Artificial Intelligence Chatbots Based on the WHO Global Guidelines for the Prevention of Surgical Site Infection: Cross-Sectional Study
Source: J Med Internet Res. 2025 Jul 31;27:e75567. doi: 10.2196/75567 (PMC12313333; doi:10.2196/75567)

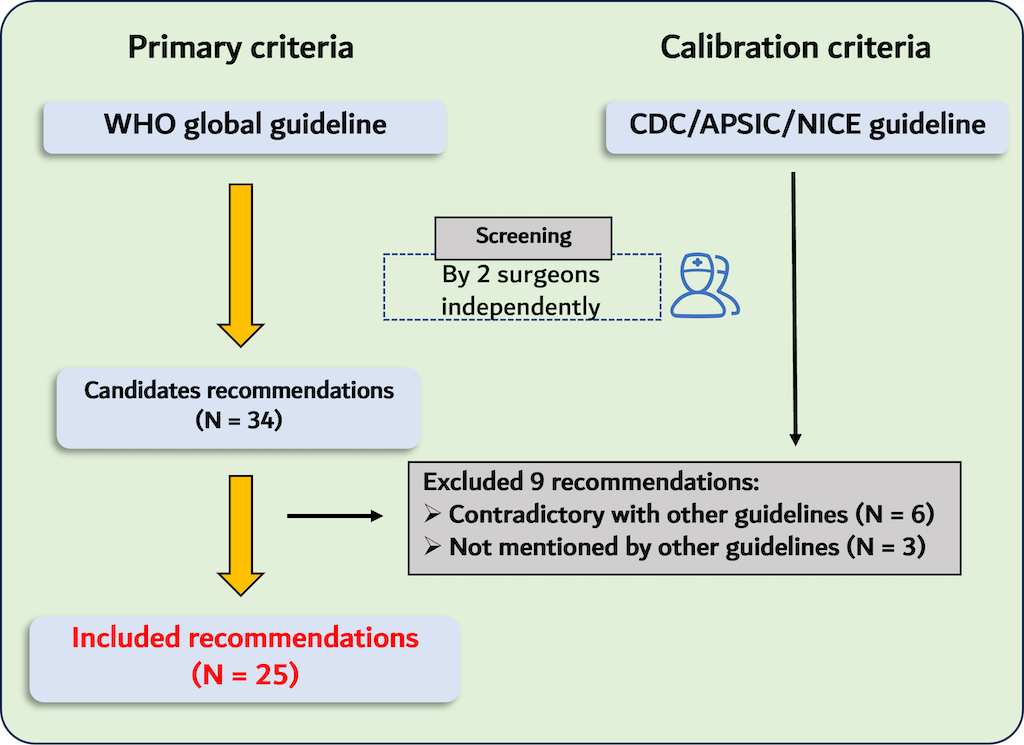

Supplement: Multimedia Appendix 2 [file jmir-v27-e75567-s002.png]
