# Supplementary material for: Evaluating the Performance of State-of-the-Art Artificial Intelligence Chatbots Based on the WHO Global Guidelines for the Prevention of Surgical Site Infection: Cross-Sectional Study
Source: J Med Internet Res. 2025 Jul 31;27:e75567. doi: 10.2196/75567 (PMC12313333; doi:10.2196/75567)
Supplement: Multimedia Appendix 3 [file jmir-v27-e75567-s003.docx]

**Multimedia Appendix 3.** The main features of the four state-of-the-art LLMs used in this study.

**ChatGPT-4o (*OpenAI*)**

ChatGPT-4o was launched on May 13, 2024, with a training data cutoff in 2023. Manufacturers claim that GPT‑4o is their latest step in pushing the boundaries of deep learning, this time in the direction of practical usability [1]. ChatGPT-4o is designed to offer enhanced capabilities over its predecessor, ChatGPT 4.0, potentially providing more accurate and contextually appropriate responses. The improvements in ChatGPT-4o include better understanding of more complex queries and improved contextual awareness [2,3]. These advancements suggest that ChatGPT-4o could offer significant benefits over ChatGPT 4.0 and the ability to potentially generate more tailored recommendations.

**OpenAI-o1 (*OpenAI*)**

OpenAI-o1 was launched on September 12, 2024, with a training data cutoff in 2023. Manufacturers claim that OpenAI-o1 can reason through complex tasks and solve harder problems than previous models in science, coding, and math. Furthermore, through training, it learns to refine its thinking process, try different strategies, and recognize its mistakes [4]. These enhanced reasoning capabilities may be particularly useful for complex problems in the medical field.

**Claude 3.5 Sonnet (*Anthropic*)**

Claude 3.5 Sonnet was launched on June 21, 2024, with a training data cutoff in 2024. It is the first frontier AI model to offer computer use in public beta. The upgraded Claude 3.5 Sonnet delivers across-the-board improvements over its predecessor, with particularly significant gains in coding, planning, reasoning, and problem-solving compared to the previous version. The Browser Company, in using the model for automating web-based workflows, noted Claude 3.5 Sonnet outperformed every model they’ve tested before [5].

**Gemini 1.5 Pro (*Google*)**

Gemini 1.5 Pro was launched on May 15, 2024. It is a new generation of AI-LLMs released by Google with a context window of millions of tokens capable of comprehending long texts, audio, and videos. Furthermore, it specializes in logical reasoning and code generation, and accessing up-to-date web information [6]. Specifically, Gemini 1.5 Pro can process large amounts of data at once, including 2 hours of video, 19 hours of audio, codebases with 60,000 lines of code, or 2,000 pages of text [7].

**References**

1. Hello GPT-4o. Published May 13, 2024. Accessed May 10, 2025. [https://openai.com/index/hello-gpt-4o/]
2. Mu LJ, Wang TT, Miao YD. Advancements in AI-driven oncology: assessing ChatGPT's impact from GPT-3.5 to GPT-4o. Int J Surg 2025;111(1):1669-1670. [doi: 10.1097/JS9.0000000000001989]
3. Zhang N, Sun Z, Xie Y, Wu H, Li C. The latest version ChatGPT powered by GPT-4o: what will it bring to the medical field? Int J Surg 2024;110(9):6018-6019. [doi: 10.1097/JS9.0000000000001754]
4. Introducing OpenAI o1-preview. Published September 12, 2024. Accessed May 10, 2025. [https://openai.com/index/introducing-openai-o1-preview/]
5. Introducing computer use, a new Claude 3.5 Sonnet, and Claude 3.5 Haiku. Published October 22, 2024. Accessed May 11, 2025. [https://www.anthropic.com/news/3-5-models-and-computer-use]
6. Liu R, Liu J, Yang J, Sun Z, Yan H. Comparative analysis of ChatGPT-4o mini, ChatGPT-4o and Gemini Advanced in the treatment of postmenopausal osteoporosis. BMC Musculoskelet Disord 2025;26(1):369. [doi: 10.1186/s12891-025-08601-3]
7. Google AI for Developers. Latest updated September 2024. Accessed May 11, 2025. [https://ai.google.dev/gemini-api/docs/models#gemini-1.5-pro]
