# Supplementary material for: Evaluating the Performance of State-of-the-Art Artificial Intelligence Chatbots Based on the WHO Global Guidelines for the Prevention of Surgical Site Infection: Cross-Sectional Study
Source: J Med Internet Res. 2025 Jul 31;27:e75567. doi: 10.2196/75567 (PMC12313333; doi:10.2196/75567)
Supplement: Multimedia Appendix 4 [file jmir-v27-e75567-s004.docx]

**Multimedia Appendix 4.** Interpretation of the 5-point Likert scales system for evaluating response of LLMs.

| QUEST dimensions | Corresponding items in this study | 5-point Likert scales | |
| --- | --- | --- | --- |
| **Accuracy**:  Correctness of response provided by the LLM. | **Accuracy** ^a,b^:  Correctness of the LLM responses compared to the benchmarks. | 5 | Completely accurate |
|  |  | 4 | Accurate |
|  |  | 3 | Neutral |
|  |  | 2 | Inaccurate |
|  |  | 1 | Completely inaccurate |
| **Relevance:**  Alignment of response provided by the LLM to the user’s query. | **Relevance** ^b^:  Correlation of the LLM responses with the prompts. | 5 | Completely relevant |
|  |  | 4 | Relevant |
|  |  | 3 | Neutral |
|  |  | 2 | irrelevant |
|  |  | 1 | Completely irrelevant |
| **Currency:**  Timeliness of response provided by the LLM. | N/A ^c^ | N/A | |
| **Comprehensiveness:**  Completeness of response provided by the LLM. | **Comprehensiveness** ^b^:  Completeness and coverage of the LLM responses to the prompts. | 5 | Extremely comprehensive |
|  |  | 4 | Comprehensive |
|  |  | 3 | Neutral |
|  |  | 2 | Incomprehensive |
|  |  | 1 | Extremely incomprehensive |
| **Consistency:**  Stability and uniformity of responses across similar queries. | **Consistency** ^a,b^:  Stability and uniformity of the LLM responses to repeat questions. | 5 | Completely consistent |
|  |  | 4 | Consistent |
|  |  | 3 | Neutral |
|  |  | 2 | Inconsistent |
|  |  | 1 | Completely inconsistent |
| **Agreement:**  Coherence of response with established facts and theories. | N/A ^d^ | N/A | |
| **Usefulness:**  Applicability and utility of the response. | N/A ^e^ | N/A | |
| **Understanding and reasoning:**  Ability of the LLM to interpret the user’s query correctly, and capability of the LLM to apply logical processing to generate the response. | **Understanding and reasoning** ^b^:  Capability of the LLM to grasp the prompts and generate the response logically. | 5 | Perfect |
|  |  | 4 | Acceptable |
|  |  | 3 | Neutral |
|  |  | 2 | Poor |
|  |  | 1 | Unacceptable |
| **Clarity:**  Quality of the response is clear, understandable, and  straightforward, making it easy for the user to comprehend the provided response. | **Clarity** ^b^:  Readability and intelligibility of the LLM responses for the readers. | 5 | Completely clear |
|  |  | 4 | Clear |
|  |  | 3 | Neutral |
|  |  | 2 | Unclear |
|  |  | 1 | Completely unclear |
| **Empathy:**  Ability of the LLM to generate a response that recognizes and reflects the emotions or tone conveyed in the user’s input, simulating a considerate and understanding interaction. | N/A ^f^ | N/A | |
| **Bias:**  Presence of systematic prejudices in the response, such as racial or gender bias. | N/A ^g^ | N/A | |
| **Harm:**  Quality of response leading to negative outcomes, such as spreading misinformation, reinforcing stereotypes, or otherwise adversely affecting users. | **Harm** ^a,b^:  Tendency of the LLM responses contributing to negative outcomes or adverse effect. | 5 | Completely harmless |
|  |  | 4 | Harmless |
|  |  | 3 | Neutral |
|  |  | 2 | Harmful |
|  |  | 1 | Extremely harmful |
| **Self-awareness:**  An LLM does not possess self-awareness in the human sense; this quality refers to the LLM’s capability to recognize its processing patterns and limitations. | **Self-awareness** ^h^:  The ability of LLM to recognize its limitations and avoid overconfidence. | 5 | Perfectly self-aware |
|  |  | 4 | Self-aware |
|  |  | 3 | Neutral |
|  |  | 2 | Unaware |
|  |  | 1 | Extremely unaware |
| **Fabrication and Falsification:**  (a) Fabrication is when the response contains entirely made-up information or data and includes plausible but non-existent facts in response to a user’s query.  (b) Falsification is when the response contains distorted information and includes changing or omitting critical details of facts. | **Fabrication and Falsification** ^b^:  Existence of made-up or distorted information in the LLM responses. | 5 | Perfect |
|  |  | 4 | Acceptable |
|  |  | 3 | Neutral |
|  |  | 2 | Poor |
|  |  | 1 | Unacceptable |
| **Trust and confidence:**  Confidence in the LLM that it will provide accurate, fair, and safe responses. The LLM meets or exceeds the expectations of the user in terms of response quality, relevance, and interaction experience. | **Trust and Confidence** ^h^:  The degree of trust and satisfaction for users to LLM and its responses. | 5 | Completely trust and satisfied |
|  |  | 4 | Trust and satisfied |
|  |  | 3 | Neutral |
|  |  | 2 | Distrust and dissatisfied |
|  |  | 1 | Completely distrust and dissatisfied |

^a^ indicates evaluation items for recommendation.

^b^ indicates evaluation items for rationale.

^c^ This study investigated the performance of current LLMs in providing specialized medical guidance for the prevention of SSI. Currency is an indicator that could not be evaluated and is a limitation of this study due to referencing the 2018 WHO global guideline.

^d^ Accuracy, relevance, and comprehensiveness already provide a robust evaluation of the coherence between responses and reference standards. Additionally, understanding and reasoning reflect the logical consistency of the responses.

^e^ The recommendations in the 2018 WHO global guideline have clear clinical significance. Furthermore, the recommendation prompts were designed as closed-ended questions, while the usefulness metric is more suitable for evaluating open-ended medical advice targeting specific diseases. Therefore, we believe that evaluating this metric in the context of this study design is unnecessary.

^f^ Empathy is more suitable for evaluating the responses generated by LLMs when patients seek medical advice. Considering the patients' medical conditions, LLMs should better reflect empathy to ensure their responses are more friendly and aligned with medical ethical standards.

^g^ It is important to note that the WHO global guideline is universally applicable, and these preventive recommendations are intended for patients worldwide. Therefore, the prompts used in this study are designed to be framed neutrally and do not contain specific demographic inferences.

^h^ indicates evaluation items for whole response.
