# Supplementary material for: Evaluating the Performance of State-of-the-Art Artificial Intelligence Chatbots Based on the WHO Global Guidelines for the Prevention of Surgical Site Infection: Cross-Sectional Study
Source: J Med Internet Res. 2025 Jul 31;27:e75567. doi: 10.2196/75567 (PMC12313333; doi:10.2196/75567)
Supplement: Multimedia Appendix 6 [file jmir-v27-e75567-s006.docx]

**Multimedia Appendix 6.** Comparison of the performances of chatbots’ responses for surgical site infection.

| Evaluation dimension | ChatGPT-4o | OpenAI-o1 | Claude 3.5 Sonnet | Gemini 1.5 Pro |
| --- | --- | --- | --- | --- |
| Recommendation |  |  |  |  |
| Accuracy | 4.29 ± 0.76 ^d^ | 4.14 ± 1.01 ^d^ | 4.30 ± 0.82 ^d^ | 3.40 ± 1.39 ^a,b,c^ |
| Consistency | 4.20 ± 0.60 | 3.96 ± 0.97 ^c^ | 4.32 ± 0.74 ^b,d^ | 3.80 ± 1.01 ^c^ |
| Harm | 4.57 ± 0.61 ^d^ | 4.44 ± 0.83 ^d^ | 4.42 ± 0.79 ^d^ | 3.74 ± 1.40 ^a,b,c^ |
| Rationale |  |  |  |  |
| Accuracy | 3.91 ± 0.71 ^d^ | 3.79 ± 0.73 ^d^ | 4.07 ± 0.64 ^d^ | 3.17 ± 1.20 ^a,b,c^ |
| Relevance | 4.20 ± 0.83 | 4.24 ± 0.74 | 4.27 ± 0.66 | 3.89 ± 1.01 |
| Comprehensiveness | 3.74 ± 0.75 ^c^ | 3.78 ± 0.77 ^c^ | 4.28 ± 0.62 ^a,b,d^ | 3.61 ± 0.99 ^c^ |
| Consistency | 4.01 ± 0.58 | 3.80 ± 0.85 ^c^ | 4.11 ± 0.58 ^b^ | 3.83 ± 0.79 |
| Understanding and reasoning | 4.18 ± 0.81 ^d^ | 4.12 ± 0.91 ^d^ | 4.18 ± 0.70 ^d^ | 3.67 ± 1.13 ^a,b,c^ |
| Clarity | 4.17 ± 0.83 ^d^ | 3.97 ± 0.85 ^d^ | 4.03 ± 0.80 ^d^ | 3.57 ± 0.98 ^a,b,c^ |
| Harm | 4.49 ± 0.60 ^d^ | 4.23 ± 0.89 | 4.41 ± 0.77 ^d^ | 3.75 ± 1.31 ^a,c^ |
| Fabrication and falsification | 4.38 ± 0.75 ^d^ | 4.10 ± 1.10 ^d^ | 4.38 ± 0.69 ^d^ | 3.17 ± 1.25 ^a,b,c^ |
| Whole response |  |  |  |  |
| Self-awareness | 4.03 ± 0.75 ^b,d^ | 3.58 ± 0.81 ^a,c^ | 4.30 ± 0.64 ^b,d^ | 3.45 ± 1.04 ^a,c^ |
| Trust and confidence | 4.09 ± 0.67 ^d^ | 3.85 ± 0.78 ^c,d^ | 4.25 ± 0.70 ^b,d^ | 3.32 ± 1.12 ^a,b,c^ |

^a^ indicates significant difference (*P* < 0.05) with ChatGPT-4o in Bonferroni post-hoc test.

^b^ indicates significant difference (*P* < 0.05) with OpenAI-o1 in Bonferroni post-hoc test.

^c^ indicates significant difference (*P* < 0.05) with Claude 3.5 Sonnet in Bonferroni post-hoc test.

^d^ indicates significant difference (*P* < 0.05) with Gemini 1.5 Pro in Bonferroni post-hoc test.
